# Analytical Sigma metrics: A review of Six Sigma implementation tools for medical laboratories

**DOI:** 10.11613/BM.2018.020502

**Published:** 2018-06-15

**Authors:** Sten Westgard, Hassan Bayat, James O Westgard

**Affiliations:** 1 Westgard QC, Madison, USA; 2 Immunogenetics Research Center, Mazandaran University of Medical Sciences, Sari, Iran

**Keywords:** Six Sigma metrics, quality control frequency, OPSpecs chart, method decision chart, Westgard rules

## Abstract

Sigma metrics have become a useful tool for all parts of the quality control (QC) design process. Through the allowable total error model of laboratory testing, analytical assay performance can be judged on the Six Sigma scale. This not only allows benchmarking the performance of methods and instruments on a universal scale, it allows laboratories to easily visualize performance, optimize the QC rules and numbers of control measurements they implement, and now even schedule the frequency of running those controls.

## The background behind the adoption of the Sigma metrics

We have emerged from a period of debate over the fundamental approach to analytical quality in laboratory testing. When the 2014 Strategic Conference on Quality Specifications convened in Milan, one of the provocative assignments was to determine whether total error should be improved, or even if it should continue to exist (*1*).

What followed was a prolonged discussion, heated at times, both in committee and in the literature, about the utility, practicality, and metrological correctness of measurement uncertainty (MU) *vs.* total analytical error (TEa) (*2*-*6*). That these models were placed in opposition to one another was neither inevitable nor necessary – the two models each have their strengths and weaknesses and can be of use at different points in the development, manufacturing and ultimate implementation and use of an analytical method (*7*).

After two years of back and forth, what emerged was neither a winner, nor a loser, but a *détente*. The published report of the Task and Finish Group on “Total error” (TFG-TE), published in 2017, represents an important compromise (*8*). The report notes the weaknesses of the current total error (TE) approach, as well as shortcomings of the biological variation database and the imperfect calculation of allowable total error based on biological variation data. It suggests that, in the future, measurement uncertainty will emerge as the dominant model for laboratory testing, while conceding that in the near term, laboratories will continue to apply allowable analytical TE.

This compromise coincides with an equally important moment: the rest of the world’s laboratories are voting with their feet, their wallets, and their routine operating protocols to continue to implement and even expand the total error approach and the additional technique of calculating Sigma metrics based on those performance specifications (*9*).

The debate between TE and MU serves as the prelude to the discussion of Six Sigma. Six Sigma, and Sigma metrics, continue to be applied by more and more laboratories, external quality assurance (EQA) programs, software vendors, and manufacturers, not because there is any regulatory or accreditation mandate, but simply because it is useful. When laboratories find a tool that helps them, they use it more. Therefore, it is with Sigma metrics.

Individual laboratories are not the only implementers of Sigma metrics. Most significantly, the International Federation of Clinical Chemistry and Laboratory Medicine (IFCC) working group on HbA1c standardization recommends that laboratories selecting a new HbA1c method should use the Sigma metrics to assess and judge the quality of that method (*10*). That the official task force on one of the world’s most standardized and utilized methods found Sigma metrics to be a meaningful technique for assessment and method selection speaks volumes.

## The shortest, most succinct summary of Six Sigma

Six Sigma is about defects, that is to say, errors, or, if we translate into International Organization for Standardization (ISO) terminology, non-conformances (*11*). For the laboratory, we are more comfortable thinking about false positives, false negatives, and outliers as the manifestations of our errors. Six Sigma is a technique to quantify – and then minimize – those defects.

Six Sigma started decades ago in Motorola and General Electric and was adopted whole-heartedly in many industries, particularly Japanese manufacturing (*12*). The terminology and amusing name conventions (green belt, black belt, master black belt, champion, *etc.*) all derive from extensive experience and success realized in other fields. In medical laboratories, the first paper to express our processes on the Sigma scale was only published in the year 2000 (*13*). Thus, the entire field of Six Sigma applications within the medical laboratory is less than two decades old.

The “Sigma” in Six Sigma refers to the benchmarking scale upon which all process defects are judged. The “Six” in Six Sigma refers to the ideal ultimate goal of all processes that six standard deviations can fit within the defined tolerance limits of a process, and that anything beyond those tolerance specifications is considered a defect. Defects can be counted or estimated and then converted to a defects-per-million (DPM) ratio (Table 1). This DPM ratio then converts into a Sigma metrics. The eponymous Six Sigma represents, on a short-term scale, just 3.4 defects *per* million opportunities, that is, *per* million times of running a process. Thus, a Six Sigma analytical method is one that is expected to generate less than four erroneous results *per* million test reports. It is a laudable goal indeed, one that is at least an order of magnitude better than most common laboratory expectations of quality. However, as laboratories have become to resemble high volume automated factories, they are producing millions of results, and the same standards of manufacturing need to apply to the standards of medical laboratory testing (*14*).

**Table 1:** Defects-per-million and the corresponding Sigma metrics

**DPM**	**Short term Sigma**	**Long term Sigma**	**Yield**
**3.4**	6	4.5	99.99966
**32**	5.5	4	99.9968
**233**	5	3.5	99.98
**1350**	4.5	3	99.87
**6210**	4	2.5	99.4
**22,750**	3.5	2	97.7
**66,807**	3	1.5	93.3
**158,655**	2.5	1	84.1
**308,538**	2	0.5	69.1
**500,000**	1.5	0	50.0
**691,462**	1	-0.5	30.9
**841,345**	0.5	-1	15.9
**933,193**	0	-1.5	6.7
Short-term Sigma is the most commonly-used and cited metric, and it assumes there is a 1.5 SD shift that occurs as the “natural” variation in a process over the long-term operation of a process. The long-term Sigma metrics does not assume any 1.5 SD shift.			

## The unique challenge of laboratory testing for Six Sigma – why we calculate rather than count a Sigma metrics for analytical methods

When a laboratory test result is generated, there is a number (or a qualitative assessment) that feels concrete, precise, and may include many decimal places. However, how do we know that number is in fact the correct, true value? In the absence of any other information, a single test result can only be assumed to be correct; to verify its truth, replicates and other comparisons against reference methods need to be done. If we do not perform this additional effort on each result, we must assume that the test result is correct based on what we know about the state of the quality control (QC), calibration and instrument performance (*15*).

Contrast that challenge with the determination of turn-around-times (TAT), which is an indicator obsessively tracked by most laboratories. It is quite simple to know whether a test result is beyond the acceptable TAT. You have the desired TAT, you have the actual TAT, and you simply count the number of times the actual TATs exceed that desired TAT. If you know what percent of your test results fail your TAT, you can convert that into a DPM, which in turn becomes a Sigma metric (*14*).

To understand the analytical method performance on the Sigma metrics scale, we do not have an easy comparative value against which we judge the test result. So instead, we leverage the data we are already collecting: imprecision (expressed as coefficient of variation, CV) and trueness (expressed as bias). This is data we routinely calculate through the use of internal quality controls, in the case of imprecision, and through either EQA or peer group programs or, rarely, through direct comparison to reference materials or methods (*16*).

While we already possess the data we need to calculate a Sigma metric, the missing piece for many laboratories is the clinical or analytical context. In order to know how well a method performs, we need to set, in industrial terms, tolerance limits: a process that exceeds its tolerance limits is generating a defect. In laboratory parlance, we need something more familiar, an allowable (analytical) total error (most commonly referred to as a TEa). When the difference between a result and its corresponding true value exceeds the TEa, that is an outlier, an erroneous result. It is a medically significant difference from the true value of what is happening with that patient (*17*).

Once we have the TEa, we have our Sigma metric (SM) equation: SM = (TEa% – bias%) / CV. This form of the equation assumes all variables will be expressed in percentages, and the bias will be an absolute percentage (the presence of any bias always shrinks the allowable error, never enlarges it). An equation can also be constructed that uses all variables in their actual units (SM = (TEa – bias) / SD) but the percentage version of the equation is most popular.

**Figure 1:**
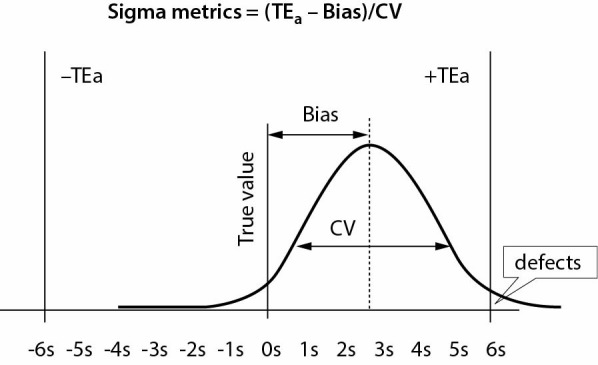
The Sigma metrics equation and a graphic description of the workings of the equation. TEa is the allowable total error (in industrial terms, tolerance limits), beyond which all results are considered defects. The bias observed shifts distribution of test results away from the “true value” of the patient. The observed coefficient of variation (CV) shows the spread of the distribution of test results. The combination of bias and CV informs where the distribution of results will occur, allowing the laboratory to estimate how many defects are produced per million results. As noted earlier, the “six” in Six Sigma comes from the designation that if six standard deviations of the process can be contained within the tolerance specifications, then less than four defects per million results will be generated.

The graphic in Figure 1 is a simple representation of how the Sigma metric equation works. The “true value” that represents the patient’s real clinical status is in the centre. As we run tests on this patient, we never get all of the results to be exactly the same, they take on the form of a normalized distribution around that value instead. The TEa are the tolerance limits on either side. If we can squeeze six standard deviations of our analytical method distribution within that TEa, in the absence of any bias, we will achieve that “Six Sigma” goal and expect to generate fewer than four clinical defective results. When bias exists, it shifts our distribution away from the patient’s true value. On the other hand, when we have a larger and larger imprecision, it spreads our distribution wider and wider. The combined impact of imprecision and bias may cause the thicker parts of the tails of our distribution to exceed the TEa, which means we are generating more defective test results (*18*).

## What is the right goal for analytical methods?

One of the unresolved issues for Sigma metrics, total allowable error, and even measurement uncertainty, is identifying the most appropriate performance specifications, goals or tolerance limits. As the literature has long noted, the goals of different regulatory systems, countries, and EQA programs are not the same (*19*). If you observe Table 2, and examine chloride, for instance, the Clinical Laboratory Improvement Amendments (CLIA) and College of American Pathologists (CAP) Survey goal is 5%. In Australia that goal (through the Royal College of Pathologists of Australasia, RCPA) is 3 mmol/L if the concentrations is less than 100 mmol/L, and 3% if the concentration is above 100 mmol/L; while the guidelines of the German medical association for the quality assurance of laboratory medical examinations (RiliBÄK) interlaboratory comparison goal is 8%. Does this mean clinical treatment is more than twice as good in Australia or nearly three times worse in Germany? Unfortunately, all these discrepancies mean right now is that there is no standardization nor harmonization of the existing resources for TEa goals. As Graham Jones and the European Federation of Laboratory Medicine (EFLM) Task and Finish Group on Specifications for External Quality Assurance Schemes (TFG-APSEQA) have noted, some EQA programs are “educational” without any penalty, so they tend to have tighter goals, while other regulatory programs have severe financial punishments for laboratories that fail to achieve these TEa goals, and thus their TEa’s are wider and more permissive (*20*, *21*). It should be noted that these differences have always existed, since the beginning of laboratory medicine and the founding of EQA surveys. In 1999, a “Stockholm Consensus” emerged that ranked the different types of TEa goals into five distinct levels of desirability, as a way to nudge laboratories toward using better performance specifications (*22*). However, as more and more laboratories implement Sigma metrics, it has become clear that the differences between TEa goals have significant impact on laboratory operational routines.

**Table 2:** Selected examples of variability in performance specifications from different sources

**Analyte**	**RCPA-QAP**	**“Ricos Goal”, desirable TEa**	**RiliBÄK**	**CLIA**
Sodium	3 mmol/L ≤ 150 mmol/L; 2% > 150 mmol/L	0.9%	5%	4 mmol/L
Potassium	± 0.2 mmol/L ≤ 4.0 mmol/L; 5% > 4.0 mmol/L	6%	8%	0.5 mmol/L
Chloride	3 mmol/L ≤ 100 mmol/L; 3% > 100 mmol/L	1.5%	8%	5%
Calcium	0.1 mmol/L ≤ 2.5 mmol/L; 4% > 2.5 mmol/L	2.4%	10%	0.25 mmol/L
Cholesterol	0.3 ≤ 5.00 mmol/L; 6% > 5.00 mmol/L	8.5%	13%	10%
Triglycerides	0.20 ≤ 1.60 mmol/L; 12% > 1.60 mmol/L	28%	16%	25%
RCPA-QAP - The Royal College of Pathologists of Australasia quality assurance programs, Australia. TEa – total allowable error from biologic variation. RiliBÄK - guidelines of the German medical association for the quality assurance of laboratory medical examinations. CLIA - Clinical Laboratory Improvement Amendments, USA. Adapted from (*19*).				

This of course means there is a potential for chaos when a Sigma metric is calculated, since different laboratories and different countries may impose different goals for TEa (*17*). The Sigma metrics of a method judged in Australia may be far harsher than the judgment rendered by the use of CAP survey and US CLIA goals. Jones *et al.* noted that there is no short-term solution on the horizon to these discrepancies – the incentives for each country and EQA survey to have a distinct and proprietary set of goals outweighs the common good of standardizing to a global set of goals (*20*). Not only would it be difficult to get so many organizations to agree on a common set of TEa goals, but also that consensus would likely contribute to the end of many small EQA programs and organizations. The Milan Consensus made its most distinct and well-recognized contribution to the discussion of performance specifications to supplant the 1999 Stockholm Consensus with the 2015 Milan Consensus. This did not select “one and only one” set of performance specifications – some analytes are better suited to one model over another; indeed it stated that there is unlikely ever to be a single model of TEa goals that will address all methods and clinical uses (*23*). They did condense the 5-level Stockholm hierarchy into three more compact levels or models:

1. Clinical use
2. Biological variation
3. State of the Art.

While the consensus stated that there was no ranking in this hierarchy, despite the numbering, it is clear the heads of the Milan committee felt that setting performance specifications based on the actual clinical use of the test results remains the gold standard. It is, however, the rarest type of performance specification – often only available for a few analytes with very specific narrow clinical uses. Thus, HbA1c, with its decades-long standardization effort by National Glycohemoglobin Standardization Program (NGSP) and CAP, enforced by tightening TEa goals, have really forced the diagnostic industry to improve methods to achieve a performance specification that supports an accurate diagnostic of diabetes at 6.5% HbA1c. Nevertheless, there are not many other analytes where these goals have been so well-stated and supported.

That leaves the second model, performance specifications based on the knowledge of within-subject biological variation. Starting in 1999, these TEa goals were continuously assessed, updated, and expanded by a group of EQA scientists in Spain lead by (now-retired) Dr. Carmen Ricos (for many laboratories around the world, these TEa goals are known as “Ricos goals”) (*24*). This burgeoning database reached more than 350 analytes at its peak in 2014, but the Milan Consensus noted many structural and methodological weaknesses (*25*-*27*). Committees are now in the process of restating these Ricos goals based on more rigorous, recent research, with all results thus far generating smaller TEa goals than the original database (*28*, *29*). One of the dangers of these new goals is that they may generate TEa goals so small that no method on the market may be able to achieve the targets. Indeed, the Milan Consensus acknowledged this and one of the ironic outcomes of the restating of these Ricos goals may be that they become so small as to be practically irrelevant to laboratory practice. If TEa goals are shrunk so minute such that no manufacturer can provide a method that achieves the desired performance, those goals are no longer practical tools, but instead are, at best, future guidelines for the next generation of research and design. Recently the EFLM task and finish group on biological variation published an appraisal checklist to enable standardized assessment of existing and future publications of biological variation data. The checklist identifies key elements to be reported in studies to enable safe, accurate and effective transport of biological variation data sets across healthcare systems. The checklist is mapped to the domains of a minimum data set required to enable this process (*30*).

Which leads us to the State of the Art, a category the Milan Consensus now defines to include all current performance goals set by CLIA, CAP, RiliBÄK, RCPA and any other existing EQA surveys (*31*-*34*). While these are the least evidence-based goals in terms of clinical need, they still represent the vast majority of goals that are implemented and used throughout the world. Therefore, while the Milan Consensus condensed the hierarchy, it specifically granted laboratories the freedom to select the most practical goals when necessary. This of course means the potential for chaos in performance specifications will continue (*35*). However, as new tests emerge, as new clinical uses for existing tests change and evolve, so too will our TEa goals. We should not expect that we will have a definitive answer in perpetuity for any test – we will always have something to discuss.

For Sigma metrics, this means that laboratories reviewing the results of these calculations must carefully consider the TEa goals applied. Many of the studies published tend to apply either CLIA or the “old Ricos” goals when calculating Sigma metrics. Our own most recent efforts through Sigma Verification of Performance is to impose a global set of TEa goals – derived from all performance specification resources - on the laboratories seeking a third-party confirmation of their performance. So even if our performance specifications will never be static over the long run, in the short term we can achieve a consensus of sorts, simply by stepping outside the silos of national regulations and EQA programs, and through an approach pioneered by Ricos and several Spanish EQA programs working cooperatively, determine a minimum global consensus for TEa (*36*).

## Beyond calculations: Visualizing world class quality

Calculating the Sigma metrics, while challenging, can be accomplished. Nevertheless, if you ask laboratories whether they want one more statistic to calculate and add to their files, you will not be surprised if the initial response is a pleading for simplicity, not more numbers.

**Figure 2:**
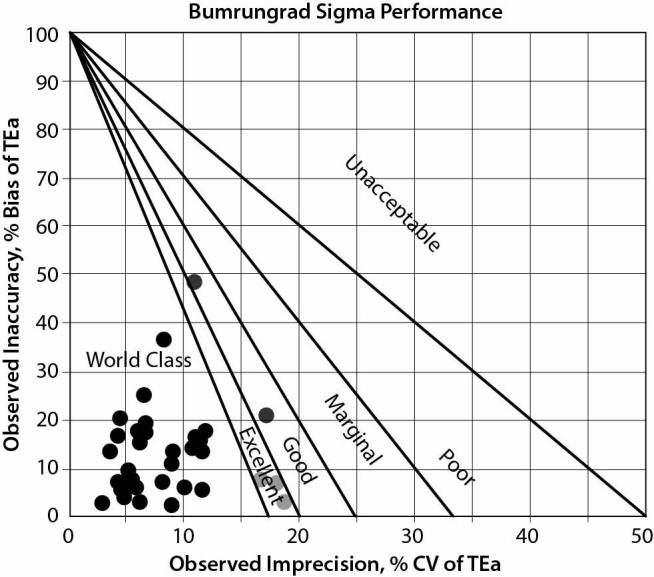
Example of a Sigma Method Decision Chart. Inaccuracy (bias, trueness) is the y-axis. Imprecision (CV) is the x-axis. This is a normalized Method Decision chart, which means multiple analytes with different TEa’s can be displayed on a single chart. This particular chart plots the performance of 32 biochemistry methods from the laboratory of Bumrungrad International Hospital in Bangkok, Thailand (*38*, *39*). The majority of plotted performance falls in the World Class or Six Sigma zone, with 3 assays in the Excellent or Five Sigma zone, and 2 assays in the Good or Four Sigma zone. There are no tests that performance at 3, 2 or less than 2 Sigma on this chart.

There is a visual tool that converts all the Sigma metrics into a simple intuitive dashboard. It is called the Method Decision Chart and it takes all the information in the equation and renders it into a graphic format (*37*). Often called a Sigma “Bull’s-Eye” graph, this chart arranges the imprecision along the x-axis and bias along the y-axis, thus the performance data from an analytical method transforms into graph coordinates (Figure 2). Superimposed on this graph are the Sigma metrics zones, with world class quality (Six Sigma), the zone closest to the graph’s origin, followed by a Five Sigma zone (Excellent), Four Sigma zone (Good), Three Sigma zone (Marginal), Two Sigma zone (Poor), and the rest of the graph, for Sigma metrics performance below Two Sigma, is labelled unacceptable. As methods get closer to the bull’s-eye, that means their Sigma metrics are higher and fewer defects are being generated. As methods perform further away from the bull’s-eye, they are generating more defects, adding more noise to the patient’s signal, and ultimately could be confounding and confusing the clinicians, not helping to confirm a diagnosis.

These Sigma Method Decision Charts can be generated for any individual allowable total error, or a Normalized chart can be generated, which allows all tests to be adjusted to display on a single graph. This dashboard approach allows a laboratory to get a succinct comprehensive view of an entire instrument’s performance, if they’re willing to plot all the data.

## Beyond the verdict of Six Sigma: Implications, implementation, and optimization

It is fine to know the Sigma metrics of your method. Perhaps it can identify that you have excellent methods, or even world class methods. Perhaps you can pre-emptively assess and dispense with the consideration of purchasing sup-par methods for your next instrument. However, can it change your life – and your laboratory life – right now?

If you are willing to bring to bear an additional set of tools, the answer is emphatically yes. Through the assessment of Sigma metrics, you can specify the number of control rules, the number of control materials and most recently, even the necessary frequency of running those controls. Sigma metrics are not just for ascertaining the quality of your method; they are the gateway to designing a customized, optimized QC strategy for the method.

In order to understand the benefits of this bespoke QC design, we need to back up and review the current common QC practices of laboratories worldwide. A global survey from 2017 noted that the most common QC rules and practices remain rooted in the past: control limits set at two standard deviations, or the complete multirules applied on all tests throughout the laboratory; frequent repeating (and repeating and repeating) of controls, frequent recalibrations, disastrous self-delusions about where to set control limits (blindingly wide), and worse, sometimes the release of patient results even in the face of an out-of-control alert, a complete disregard for the quality control process itself (*40*).

Intuitively, laboratories realize that the best methods should be the most reliable, and therefore require less effort to monitor and control. Conversely, the worse methods will need the most rules, more controls, and need to have that QC run more often. The Sigma metric QC design tools help specify just how much QC effort is required based on the performance of the method and the quality required to the needs of the patient (TEa).

**Figure 3:**
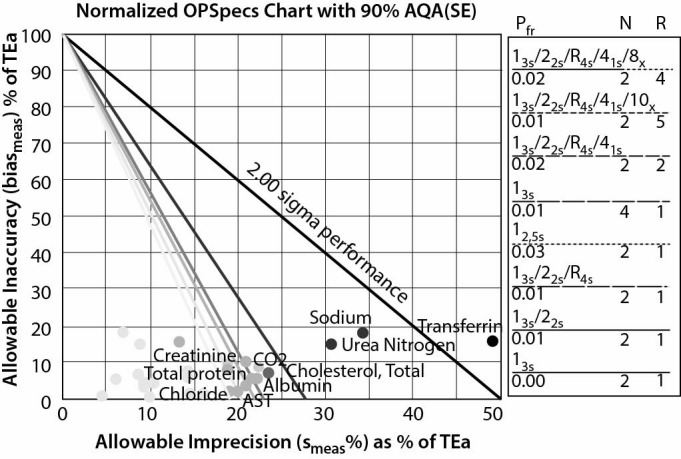
A Normalized OPSpecs chart. Inaccuracy (bias, trueness) is the y-axis. Imprecision (CV) is the x-axis. This is a normalized chart which allows multiple tests to be displayed on one chart, in this case 23 assays from an anonymous US hospital core laboratory. The diagonal lines displayed in the chart represent (in order from right to left) different quality control (QC) procedures listed in the key at right (from top to bottom). In the key, Pfr stands for probability of false rejection, N stands for the number of control measurements made, and R stands for the number of runs over which the rules are applied. As performance is closer to the “bull’s-eye”, the plotted point is “within the ring” of that QC procedure, which means that that particular rule combination will provide adequate error detection and appropriate analytical quality assurance. Note that for three of these analytes, performance is very poor and will require not only the full multirule QC procedure (all the “Westgard Rules”) but will require many more controls to be run. AQA (SE) - analytical quality assurance for systematic error. TEa - total analytical error.

The first graphic tool is called the OPSpecs chart, or chart of operating specifications (*41*). This tool details how many rules and controls are needed to provide the necessary error detection (with a minimum of false rejection) for the method. It is a graph very similar to the Six Sigma Method Decision chart, in that the imprecision forms the x-axis, and the bias forms the y-axis (Figure 3). Again, performance of a method through its CV and bias form the coordinates to plot on the chart. Where the OPSpecs chart differs is that the lines displayed on the chart are no longer Sigma metrics zones, they represent the performance of different QC procedures.

Each line in the OPSpecs chart is like one of the rings of the bull’s-eye. A method falling “within that ring of the target” can safely use the QC procedure and gain an appropriate level of analytical quality assurance. Conversely, if the operating point of method falls “outside that ring” of a QC procedure, then the use of that QC procedure will not provide adequate error detection or quality assurance. Thus, the principle of the Method Decision chart is the same for the OPSpecs chart. The closer the operating point of a method is to the graph’s origin, or bull’s-eye, the easier the QC effort is. A method that actually “hits the bull’s-eye” actually does not need to use any “Westgard Rules” at all – they can safely use wider control limits set at 3 SD or perhaps even wider, and a bare minimum number of controls. As Sigma metrics performance decreases, however, more and more rules are needed, tighter and tighter limits, and more and more controls. For tests with very low Sigma metrics, even the full “Westgard Rules” and a tripling of controls may not be enough to adequately monitor the method.

## Beyond rules and controls: Sigma metrics can define run length and QC frequency

Up until about a decade ago, QC frequency was entirely determined by rule of thumb. Or, if you will, it was planet-based QC frequency: once a day. Sometimes it was labour-based QC frequency: once a shift. But it was certainly not patient-based QC frequency, driven by the performance of the method and the quality required for the appropriate use and interpretation of the test results (*42*).

In 2008, Dr. Curt Parvin introduced a model that created the foundation necessary to create a patient-based QC frequency (*43*). It was not simple, nor was it well-understood when first published, and it remained obscure and mostly impractical for ten years. However, starting in 2016, additional scholarly work translated Parvin’s model into something practical for today’s busy laboratories. It began with Yago and Alcover, who translated the intricate equations of Parvin’s model into curves onto a nomogram (*44*). This meant that the decision on QC frequency no longer had to be calculated and understood mathematically, but could instead be simply interpreted graphically.

**Figure 4:**
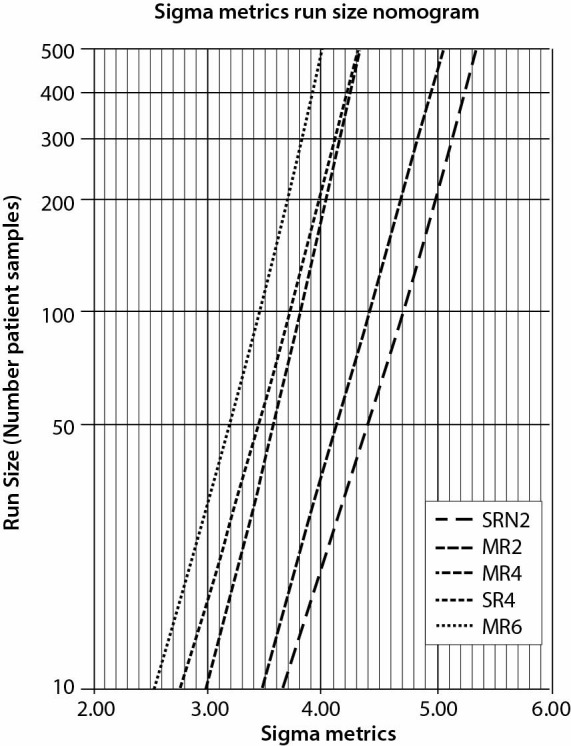
Six Sigma QC Frequency Nomogram. The Sigma metric of the method is the x-axis. The number of patient specimens that can be run in between controls is along the y-axis. The different lines in the graph represent the QC procedures that may be chosen by the laboratory. MR6, for example, represents a full implementation of “Westgard Rules” using 6 control measurements (either 6 controls run at once, or 3 controls run with 2 measurements being made on each control). SRN2 represents a set of single wide control limits (3 SD) with 2 control measurements. MR2 represents a standard set of “Westgard Rules” (multirule QC procedure) with 4 control measurements. MR4 represents a standard set of “Westgard Rules” (multirule QC procedure) with 2 control measurements. SR4 represents a set of single wide control limits (3 SD) with 4 control measurements. In order to determine QC frequency, a laboratory should first determine the method’s Sigma metric, and then find the appropriate QC design, then find the intersection of the Sigma metric and that QC procedure line. If a laboratory is lucky enough to have a Six Sigma method, for instance, it is clear that any QC procedure will do and that at least 500 patients, and possibly many more, can be run in between QC events without raising the risk of reporting a single erroneous patient result.

Through a series of papers in 2017 and 2018, Bayat, Westgard and Westgard extended these graphic simplifications to create more practical QC frequency nomograms (*45*-*48*). Now, instead of calculating esoteric variables such as Max(ENuf), laboratories can simply observe a graph that compares their Sigma metrics on the x-axis with the number of patient samples that can be run between controls on the y-axis (Figure 4). The Sigma metric directly determines how often you run QC, and for Six Sigma methods, potentially this QC frequency can be reduced to 1 control per 1000, 2000, or even higher numbers of patient samples, or they can use extended QC limits such as 4s or wider. For Three Sigma methods and lower, however, QC frequency must be greatly increased, to something closer to one control *per* 100 or *per* 50 patient samples. Methods below 3 and 2 Sigma would theoretically require almost constant running of controls, a frequency so impractical as to guarantee the dismissal of any employee daring to implement it.

## QC inaction *vs.* Six Sigma QC in action

As this review shows, Sigma metrics confirms what we intuitively understand: a poor method requires so many rules, so many controls, with controls that must be run so often, it is not practically possible to adequately monitor the method – it must be improved, redesigned, or replaced. It also confirms what we hoped was true: a world class method requires far fewer rules, controls and running of those controls than we may currently be implementing.

Laboratories have been implementing these tools for years now, and the results are encouraging. Laboratories with high Sigma metrics performance have been able to reduce their use of controls, reduce the sheer number and percentage of outliers, reduce their trouble-shooting, reduce their recalibrations, reduce even their consumption of reagent and materials (*49*-*52*). In addition to proven cost reductions through the implementation of Six Sigma techniques, there are important reductions in the labour effort of staff: fewer hours spent chasing down false rejections, fewer hours spent in unnecessary trouble-shooting, fewer hours on the phone with technical support.

The soft cost reductions of saved hours and improved morale are less easily observed on the budget ledger, but probably of more significant impact to the working culture and healthy morale of the laboratory.

Contrast these potential impacts with the possible impact of an implementation of MU. No commonly accepted goals for allowable uncertainty exist – most often when MU is calculated, it is compared against Ricos goals, which is a wholly unrelated quantity (*39*). No relationship between the use of QC rules, number of controls, or required frequency of QC and measurement uncertainty exist. If laboratories were to stop using Sigma metrics and TEa and confine themselves only to the calculation of MU, what would they do for QC, and numbers of control, and frequency of QC? That would be completely… uncertain.

## A footnote on the missing utility of measurement uncertainty

As laboratories around the world make progress with Sigma metrics, at the same time they are paying the merest lip service to MU. Without doubt, when coerced by inspectors and ISO requirements, laboratories calculate MU, but most will only calculate the statistic, record it in a file, which is displayed upon request to an inspector, and then promptly ignored once the inspection is finished (*9*).

As stated earlier, there is ample room for coexistence. Measurement uncertainty is particularly helpful at the manufacturing stage, where the diagnostic industry has the power and incentive to monitor, modify and minimize uncertainty. However, once the analytical method has been introduced to the marketplace, the modern nature of laboratory operations mean that technicians have little recourse to fixing unacceptable uncertainties. If they are “stuck” with a box that has too much MU, there are few ways to fix the problem other than replacing the box with something better. Again, this shows how MU can be most useful at the manufacturing level – by preventing the diagnostic industry from producing instruments that will be clinically useless once they reach the market.

## Conclusion: Don’t remain MUTE, move forward with MU and TE

The Milan Consensus supports the continued use and implementation of TEa and Sigma metrics. Rather than mire ourselves in further debate, let us make progress. Using total error, laboratories can calculate their Sigma metrics, and use tools like the Method Decision Chart, OPSpecs chart, and QC Frequency Nomogram, to implement changes in their consumption of control materials, reagent, and calibrators, in the interpretation of control rules, and in the frequency of running controls. All of these actions, when world class quality methods are in place, drive lower costs and less effort in the laboratory.
